# Use of Parsing Heuristics in the Comprehension of Passive Sentences: Evidence from Dyslexia and Individual Differences †

**DOI:** 10.3390/brainsci12020209

**Published:** 2022-02-01

**Authors:** Marianna Stella, Paul E. Engelhardt

**Affiliations:** 1 School of Social Sciences and Humanities, University of Suffolk, Ipswich IP4 1QJ, UK; 2 School of Psychology, University of East Anglia, Norwich NR4 7TJ, UK; p.engelhardt@uea.ac.uk

**Keywords:** dyslexia, reading disability, language comprehension, sentence processing, passive sentences, semantic plausibility

## Abstract

This study examined the comprehension of passive sentences in order to investigate whether individuals with dyslexia rely on parsing heuristics in language comprehension to a greater extent than non-dyslexic readers. One hundred adults (50 dyslexics and 50 controls) read active and passive sentences, and we also manipulated semantic plausibility. Eye movements were monitored, while participants read each sentence, and afterwards, participants answered a comprehension question. We also assessed verbal intelligence and working memory in all participants. Results showed dyslexia status interacted with sentence structure and plausibility, such that participants with dyslexia showed significantly more comprehension errors with passive and implausible sentence. With respect to verbal intelligence and working memory, we found that individuals with lower verbal intelligence were overall more likely to make comprehension errors, and individuals with lower working memory showed particular difficulties with passive and implausible sentences. For reading times, we found that individuals with dyslexia were overall slower readers. These findings suggest that (1) individuals with dyslexia do rely on heuristics to a greater extent than do non-dyslexic individuals, and (2) individual differences variables (e.g., verbal intelligence and working memory) are also related to the use of parsing heuristics. For the latter, lower ability individuals tended to be more consistent with heuristic processing (i.e., good-enough representations).

## 1. Introduction

Research into the comprehension of passive sentences has a long history in psycholinguistics [1,2,3,4,5], and has also been looked at developmentally [6,7,8,9] and in clinical populations (e.g., aphasia). Passive sentences are interesting because they are syntactically more complex than actives and violate the canonical subject-verb-object word order in English. With passive sentences the object comes first and the subject follows the verb, and relatedly, the thematic roles are also reverse (i.e., patient/theme sentence initial and agent sentence final).

### 1.1. Good Enough Comprehension

One prominent theory that has been offered to account for the fact that listeners often develop inaccurate representations in language comprehension is called “Good Enough” processing [10,11,12]. According to this theory, listeners may generate an interpretation of an ambiguous or a temporarily ambiguous sentence that is not consistent with the actual input. Instead, the comprehension system has a tendency to generate shallow or superficial representations, and much of the time misinterpretations are consistent with the plausibility of events in the real world [13,14]. One of the main aims of the current study was to investigate whether readers with dyslexia rely on parsing heuristics (good-enough processing) to a greater extent than typically-developing individuals, and how they comprehend passive sentences more generally.

The vast majority of research on the comprehension of passive sentences has looked at whether listeners can correctly identify the thematic roles in the sentence. In one prominent study, Ferreira [15] conducted three experiments in which participants listened to sentences in active and passive voice that were either semantically plausible or implausible. Participants were asked to identify one of the thematic roles in the sentence (e.g., who was the agent in the sentence?). Ferreira’s results showed that passive sentences were misinterpreted more frequently than active sentences, and the differences were greater for passive-implausible sentences (e.g., the dog was bitten by the man.). Ferreira referred to these kinds of (passive-implausible) sentences as “biased-reversible”, because real-world semantic knowledge “biases” people to assume that the dog was the agent of the action (i.e., in the real world it is much more likely for dogs to bite men than vice versa). “Reversible” refers to the fact that both nouns in the sentence are animate, and thus capable of performing the action described by the verb.

Based on the results from her study of passives, Ferreira [15] postulated that two parsing strategies (or heuristics) underlie participants’ tendency to engage in good-enough processing. The first is a syntactically-based strategy, and referred to as the “noun-verb-noun” (NVN) strategy. This strategy assumes that comprehenders tend to assign the subject role to the first noun in the sentence (i.e., the subject is the agent of the action) and assign the object role to the final noun in the sentence (i.e., that the object is the patient or theme). This follows the highly dominant frequency bias in English for sentences to follow subject-verb-object word order. Several corpus studies report that active sentences occur approximately 99% of the time in spoken language and 95% of the time in written language [16,17]. The second strategy postulated by Ferreira [15] was referred to as the “semantic-plausibility” (SP) strategy. This strategy has participants consult their knowledge about states of affairs in the real world, and in cases where there is a conflict between sentence content and real-world knowledge, comprehenders choose the interpretation that is more likely to have occurred in the real world.

In summary, the use of strategies in comprehension results in situations in which the actual meaning of a sentence is incompatible with the participant’s interpretation of that sentence. The use of strategies in language comprehension is assumed to be an adaptive function based on fast and frugal heuristics [18,19]. The basic idea is that they permit (cognitive) short cuts that override the more time-consuming and cognitively demanding algorithmic parsing governed by the full set of grammatical knowledge held by a competent speaker. Ferreira [15] referred to these as pseudo parsing and algorithmic parsing, respectively.

**Figure 1:**
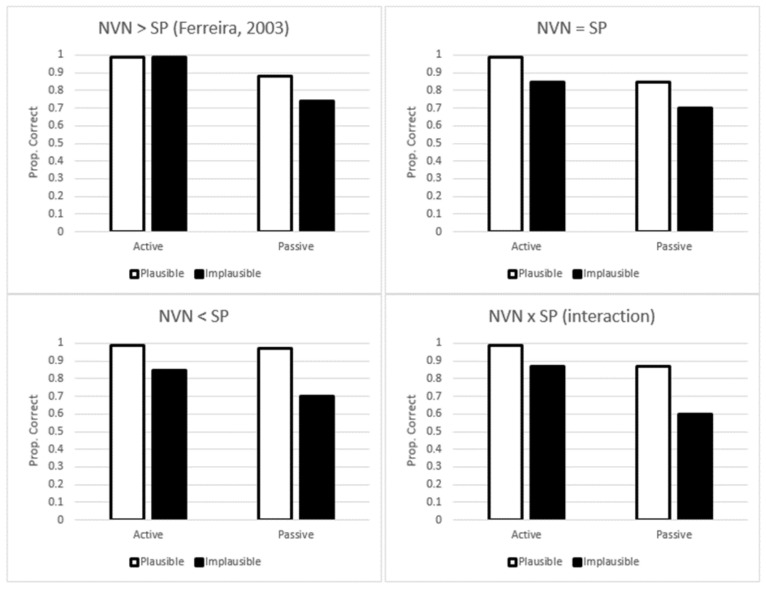
Predicted comprehension results based on the impact of noun-verb-noun (NVN) and semantic plausibility (SP) heuristics.

One question that naturally arises is how often participants adopt a good-enough interpretation based on fast-and-frugal processing strategies rather than the full algorithmic parse. Results from the Ferreira [15] study showed that listeners were equally good (and in fact, near perfect) for both active-plausible and active-implausible sentences (see Table 1). However, for passive sentences, listeners committed errors in approximately one out of every five sentences, and there was a clear difference between plausible and implausible passives. The results of Experiment 2 [15] are shown in the upper-left panel of Figure 1, and based on this pattern, Ferreira concluded that the noun-verb-noun strategy is employed more often than the semantic-plausibility strategy. In the other panels of Figure 1, other possibilities comparing the two different processing strategies with one another are shown. However, in order to be clear, we think it is important to work through these differential predictions systematically. With active-plausible sentences (the easiest of the four conditions), neither NVN or SP strategies are assumed to be employed. With active-implausible sentences, participants have the potential for misinterpretations if they go with what was more likely to have happened in the real world (semantic-plausibility). With passive-plausible sentences, participants have the potential for misinterpretations if they assign the subject role to the sentence initial noun phrase and object role to the sentence final noun phrase (noun-verb-noun). Finally, with passive-implausible sentences, the potential for misinterpretation is the highest because both strategies could be employed (i.e., this is the most difficult condition). 

Returning to the issue of how often comprehenders engage each type of strategy, Ferreira [15] concluded that noun-verb-noun was employed more than semantic plausibility. If both strategies affect comprehension equally, then we should observe a pattern like the one shown in the upper-right panel of Figure 1 (i.e., a double main effect). If semantic plausibility is employed more frequently, then the pattern should be like the one shown in bottom-left panel. Finally, if the two strategies interact with one another, then we should observe the pattern shown in the bottom-right, which would be an under-additive interaction.

### 1.2. Comprehension in Dyslexia

Studies on dyslexia have described syntactic processing deficits in both oral and written language across the lifespan [20,21]. Impairments in the comprehension of syntactically complex sentences may arise from several factors: (1) a specific linguistic deficit with respect to syntax, (2) deficits in cognitive abilities that underlie language comprehension, like working memory and/or processing speed [22,23], or (3) a secondary consequence of reduced reading experience [24]. For the latter, it is suggested that slow and laborious decoding exhausts cognitive resources, which leads to increased likelihood of decoding errors/substitutions, with an overall negative impact on sentence and text comprehension. However, there has been limited focus on investigating whether individuals with dyslexia have deficits in sentence comprehension [25,26]. This is important because many of the existing dyslexia studies have focused on single-word decoding, but there are considerable differences between reading single words and comprehending sentences.

To date, there has been only one study on the comprehension of passive sentences in individuals with dyslexia. Wiseheart, Altmann, Park, and Lombardino [27] examined sentence comprehension in adults with and without dyslexia while reading active and passive sentences. In their study, they used non-biased reversible sentences (e.g., the queen kissed the king. vs. the king was kissed by the queen), which means that there was no bias between the potential doer of the action and patient of the action. Participants were shown two images side-by-side on the computer screen under the sentence and they had to choose which picture corresponded to the sentence. Wiseheart et al. [27] showed that dyslexic readers were marginally slower in their response times and had poorer comprehension accuracy on passive sentences compared with the control group. Controls were 98% accurate on actives and 95% accurate on passives. In contrast, participants with dyslexia were 98% accurate on actives and 83% accurate on passives. In their conclusions, Wiseheart et al. [27] argued for a frequency-based (or exposure-based) explanation. Research also suggests that individuals with dyslexia are not impaired in statistical learning [28,29,30]. In general, people encounter passives much less frequently than actives, and given dyslexics’ difficulties with reading and their inherent aversion to reading, the frequency differential for people with dyslexia would be even greater [16]. 

We think this explanation contradicts what Dabrowska and Street [31] showed with regards to non-native English speakers actually performing better on the comprehension of passive sentences than native English speakers. This is because non-native speakers have less exposure compared with native speakers. In the current study, we pursued an alternative explanation for difficulties shown by individuals with dyslexia in the comprehension of passive sentences. We hypothesise that individuals with dyslexia may be more likely than typically developing readers to engage in good enough processing, and thus more likely to apply comprehension strategies (i.e., noun-verb-noun or semantic plausibility). It is also essential to highlight that multiple studies on dyslexia have shown that individuals with dyslexia, and particularly children, use context to compensate for poor word-decoding skills [32,33,34], which we expect to observe in processing of passive sentences. It is also possible that individuals with dyslexia utilise their real-world knowledge to a greater extent, to again compensate for difficulties with decoding. 

### 1.3. Individual Differences

Another aspect that we focused on in this study was individual differences in verbal intelligence and working memory. There are many studies showing an effect of working memory on sentence processing and sentence comprehension [35,36,37,38,39,40]. However, there is some debate over the strength of the relationships, particularly for online (reading times) and offline (comprehension accuracy). Previous studies have shown that individuals with dyslexia have lower working memory and slower processing speed [41,42,43,44,45]. However, in a recent study, we found that the comprehension of garden-path sentences was much more related to individual differences in working memory than processing speed [46,47]. Several recent individual differences studies have shown that the best predictor of the comprehension of syntactically complex sentences is verbal intelligence [48,49]. Thus, we thought that both working memory and verbal intelligence were the two most promising individual difference variables to investigate with respect to dyslexia and parsing heuristics. 

### 1.4. Current Study

The main aim of the current study was to investigate the comprehension of passive sentences in individuals with dyslexia. We hypothesised that individuals with dyslexia are more likely to rely on good enough processing, and thus are more likely to rely on comprehension strategies. We used the “biased-reversible” sentences (see Table 1) from Ferreira [15] because these sentences have the potential to create conflict between sentence content and real-world knowledge (i.e., these sentences are specifically the ones that tap into the semantic-plausibility strategy) [10]. Thus, the materials used in the current study were expected to show some effect of both the syntactic (noun-verb-noun) strategy and the semantic-plausibility strategy. We also monitored eye movements in order to assess how long participants read each sentence. According to the Good Enough theory, the application of parsing strategies occurs because comprehenders seek to generate interpretations, while at the same time keeping the demand on cognitive resources as low as possible [10,41]. Thus, if good enough processing is engaged, then we might expect reading times to be shorter for trials in which the participant makes a comprehension error. In other words, faster reading speeds would be associated with lower comprehension (i.e., speed-accuracy trade off consistent with good enough processing). In contrast, readers may slow down to navigate a complex sentence to ensure accurate understanding by reading slowly (and re-reading). In other words, slower reading speeds would be associated with higher comprehension (i.e., processing consistent with effortful algorithmic parsing). 

In the current study, we had two broad research objectives. The first focused on whether individuals with dyslexia rely on parsing heuristics to a greater extent than individuals without dyslexia. In general, given what is known about dyslexia, we expected individuals with dyslexia to show lower comprehension and higher reading times [27]. As mentioned previously, these issues may be due to a specific linguistic deficit, an issue associated with another (related) individual difference variable (see below for more information), or slower more laborious word decoding. However, by manipulating both structure type (active vs. passive) and plausibility (plausible vs. implausible) in biased-reversible sentences, we were also interested in assessing whether participants would use the noun-verb-noun strategy and semantic-plausibility strategy, and whether the two groups of participants (dyslexics and controls) would show the same pattern. More specifically, we wanted to investigate whether they would show one of the patterns shown in Figure 1. 

The second broad research objective focused on individual differences in verbal intelligence and working memory. Previous research has highlighted the importance of verbal intelligence and working memory in sentence processing and comprehension [46,47,48,49]. Therefore, we believed that it was important to assess and ultimately control for individual differences in both verbal intelligence and working memory. Our specific research question for this objective was how do individual differences in verbal intelligence and working memory affect both comprehension accuracy and reading times? To assess individual differences, we conducted additional analyses in which we examined verbal intelligence and working memory separately.

There is one further point worth mentioning with regards to the memory demand of the task we used. We included a maths problem in between the sentence and the comprehension question, and participants had to determine whether the maths problem was correct or not. (Participants received feedback on their response to the maths problem.) The rationale for including this additional task was that we wanted to assess the representation that comprehenders generated of the sentence without allowing them to have direct access to the sentence. We assumed that the presence of the maths problem would clear the immediate contents of memory, and thus participants would be answering the comprehension question on the basis of a more long-term and stable representation of the sentence they had just read.

## 2. Materials and Methods

### 2.1. Participants

Fifty adults with dyslexia were recruited via advertisements and 50 undergraduate psychology students were tested as control participants. Psychology students were recruited through the participant pool and received course credit. Dyslexic students were primarily recruited through disability liaison officers in different departments as a function of being on the disability register at the university. Both groups were recruited from the campus of the University of East Anglia. All participants with dyslexia verified that they had a prior diagnostic assessment for dyslexia (by an educational psychologist or dyslexia specialist) prior to study enrolment. All were native speakers of British English with normal or corrected-to-normal vision. Dyslexics were reimbursed with £16 for their time. Demographic information about the two groups is provided in Table 2. Note that the groups were not matched on age, nor gender, but correlations in the results section demonstrate that age and gender did not significantly correlate with the dependent variables. 

### 2.2. Standardised Measures

#### 2.2.1. Rapid Automatised Naming 

All participants completed both a letter and a number RAN test using the second edition of the Comprehensive Test of Phonological Processing (CTOPP 2) [50]. The RAN task requires participants to name a series of letters or numbers sequentially out loud as quickly and accurately as possible. The time taken to complete an array was recorded with a stopwatch. Participants completed one letter and one number array for practice, and two served as the critical trials (i.e., one letter array and one number array). The score for each task was the total time that was needed to complete the task; higher scores indicate worse performance. Each array consisted of four rows of nine items. Letters and numbers were presented in Arial font, and all items appeared on the same side of white A4 paper. The standardised procedures of administration for this task were followed as described in the test manual. Independent samples *t*-tests revealed significantly longer naming times for the dyslexic group compared with controls on both versions of the task (see Table 2), which is consistent with prior studies [36]. The reliability of the CTOPP-2 subtests has been demonstrated by average internal consistency that exceeds 0.80 [50].

#### 2.2.2. Working Memory 

A rotation span task was used as a measure of working memory, as it has been shown to assess both processing and storage functions [39,51]. Participants were required to look at a rotated letter and then verify whether or not the letter is facing in the correct direction or mirrored. After each letter, participants were presented with an isolated arrow which was either long or short and could be facing eight different directions (0°–360°). The position and length of the arrows presented needed to be recalled at the end of the set. The task consisted of 15 trials (six trials of each set of lists consisting of 2 items that needed to be recalled and three trials of each set of lists consisting of 3–5 items that needed to be recalled) and, in total, 48 arrow-storage pairs [51]. The rotation span task developed by Engle’s Working Memory Laboratory, and reported reliability ranges between 0.67 and 0.77 [52].

#### 2.2.3. Verbal Intelligence 

Verbal intelligence was measured by the following subtests of the fourth edition of the Wechsler Adult Intelligence Scale (WAIS-IV) [53]: vocabulary, comprehension, and similarities. In the comprehension task, participants were required to respond to questions about general concepts (e.g., reasons to protect endangered species). Vocabulary requires participants to provide the definitions of words and measures the degree to which one has learned and is able to express meanings verbally. Similarities requires participants to describe how two words are similar, with the more difficult items typically describing the opposite ends of a “unifying continuum”. The similarities subtest measures abstract verbal reasoning. For all subtests, higher values correspond to higher verbal intelligence and the score for each of these tasks was the total number of items that the participants could identify accurately. The standardised procedures of administration for these subtests were followed as described in the test manual. With respect to the reliability of the WAIS-IV, the manual reports average internal reliability coefficients for subtests that range from 0.78 to 0.94 [54].

### 2.3. Sentence Processing

We used 20 sentences, half of which were active and half were passive. Furthermore, in each category, half of the sentences were plausible and half were implausible (see Table 1). Participants also read 80 filler sentences. All filler sentences were grammatically correct and consisted of five sets of 16 sentences. The first set were subordinate-main structures in which the subordinate clause was transitive. The second set were main-subordinate sentences. The third set were transitive sentences containing a relative clause at the end of the sentence. The fourth set were transitive sentences that contained an embedded relative clause that modified the subject noun phrase. The fifth set were coordination structures, in which two transitive sentences were conjoined with “and”. Half had a comma between “and” and the preceding word, and half did not. In addition, there were also 40 sentences with relative clauses, half of which were object relative and half were subject relative. Therefore, each participant read 140 sentences in total. All 20 critical items (active and passive sentences) were rotated across two counterbalanced lists, with plausible sentences changing to implausible and vice versa (see Table 1). We assessed the length (total number of characters) and the frequency of the main content words (subject noun, matrix verb, object noun). Results showed that there were not significant length differences between the actives (Mean = 23.6, SD = 6.02) and passives (Mean = 26.7, SD = 3.27) *t* (18) = −1.43, *p* = 0.18. Analyses of frequency did show a significant difference in which the passives (Mean = 264.5, SD = 76.47) had higher frequency than did the actives (Mean = 83.5, SD = 21.71) *t* (18) = −2.28, *p* = 0.035. However, upon close inspection, this difference was driven by one high frequency outlier (i.e., the word man) in the passive condition (its frequency was 614). When this outlier was removed from the analysis, there were no longer significant differences between the conditions *t* (18) = −1.49, *p* = 0.15. Furthermore, we conducted two analyses in which we examined the effect of length and frequency on the reading time main analyses. Those results are presented in Section A of the Supplementary Materials. The comprehension questions were also rotated to match the corresponding types of sentences.

### 2.4. Apparatus

Eye movements were recorded with an SR Research Ltd. EyeLink 1000 eye-tracker (Ottawa, ON, Canada) which records the position of the reader’s eye every millisecond. Head movements were minimised with a chin rest. Viewing distance was 70 cm from eyes to a 45-cm computer monitor, and at this distance, 1.0° of visual angle subtended 1.22 cm. This apparatus allows recording of eye movements through a camera with an infrared tracking system. Eye movements were recorded from the right eye. The sentences were presented in 12 pt. Arial black font on a white background.

### 2.5. Design and Procedure

For the sentence-processing task, the design was a 2 × 2 × 2 (Sentence Type × Plausibility × Group) mixed design in which sentence type and plausibility were within subjects and group was between subjects. Participants completed three practice trials, 20 experimental trials, and 120 fillers. Trials were presented in a random order for each participant.

Participants were provided with a set of instructions that detailed the experimental procedure. They were then seated at the eye tracker and asked to respond to on-screen instructions using the keyboard. At the beginning of each trial, a message appeared asking the participant to press a button when they were ready to continue. After the participant pressed the button, they were required to fixate on a drift-correction dot. The experimenter then initiated the trial. The sentence appeared after 500 ms, and the initial letter of each sentence was in the same position, in terms of x and y coordinates, as the drift correction dot (i.e., on the left edge of the monitor and centred vertically).

The entire sentence was presented on a single line on the screen. The participant read the sentence silently and then pressed the spacebar on the keyboard. Following a delay of 500 ms, an arithmetic problem (either addition or subtraction) appeared on the screen (e.g., 45 + 67 = 112). The problem was presented for 3000 ms and was followed by a screen prompting the participant to press the green button on the keyboard if the solution was correct, or the red button if it was incorrect. After participants read the sentence, they were asked a comprehension question (see Table 1 for examples). For the reliability of the sentence-processing task, we computed split-half reliabilities. Because there were ten items in each of the within-subject conditions, we used Spearman–Brown prophecy formula-corrected coefficients [55,56]. The mean reliability was *α* = 0.68.

The testing session for each participant lasted approximately 2 h, with several breaks included between tasks to avoid fatigue. The tests were delivered in the following order for each participant: vocabulary, rotation span, comprehension, sentence processing, RAN digits, RAN letters, and similarities.

### 2.6. Data Screening and Analysis

In order to keep the analyses as straightforward as possible, we submitted the verbal intelligence subtests to a factor analysis (principal components extraction) in which we saved the retained factors as variables. The results of the factor analysis showed only one factor (eigenvalue = 1.81, accounting for ~60% of the total variance). The factor loadings were all significant and relatively uniform (vocabulary = 0.84, comprehension = 0.76 and similarities = 0.72). We used this composite (or latent) variable in our analyses examining ‘individual differences in verbal intelligence’. Working memory was only measured by the rotation span, and thus, that variable was used in analyses of working memory.

We analysed the comprehension, reading time, and regressions using Linear Mixed Effects models using R [57]. Results include *p*-values estimates from the lmerTest package. Fixed effects for sentence structure, plausibility, and group (dyslexia status) were included. The random-effects structure was maximally specified with random intercepts for participants and items [58,59]. In the event of convergence problems, the model was simplified (items then subjects) until convergence was achieved. Tukey contrasts are reported following significant interactions. For eye movements, we examined the reading times of the entire sentence by summing the fixation durations over the entire sentence, and we also examined the average number of regressions per sentence. We first report the comprehension results, and second the eye movements. For the reading times, we report total reading time, which is the sum of all fixations on the whole sentence. For regressions, we report the mean number of regressions per sentence. A regression is defined as a right-to-left eye movement in which the eyes move from one word to an earlier word in the sentence. 

## 3. Results

### 3.1. Comprehension Accuracy

**Figure 2:**
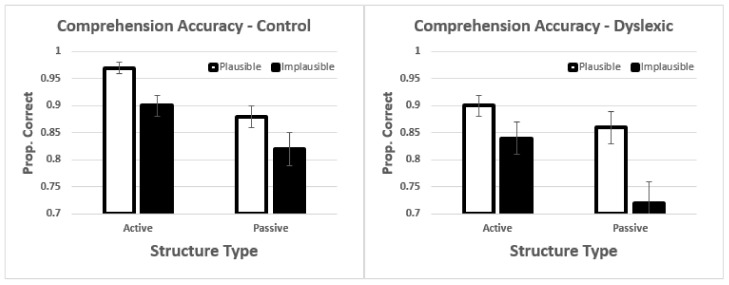
Mean comprehension accuracy. Error bars show the standard error of the mean.

For comprehension accuracy, there was a significant main effect of sentence type *t* = −3.00, *p* = 0.004 (see Figure 2). Active sentences had higher comprehension accuracy than passives. The main effects of plausibility and group were not significant (for full R output see Sections B and C, Supplementary Materials). There was a significant interaction between sentence structure and plausibility *t* = 2.46, *p* = 0.014, and a significant three-way interaction between variables *t* = −2.02, *p* = 0.04. To decompose that interaction, we examined the two-way interactions for each group separately. Controls showed a significant main effect of plausibility *t* = 2.59, *p* = 0.01, in which the implausible sentences had lower accuracy. The interaction was not significant (*p* = 0.68). Dyslexics, in contrast, showed a significant main effect of structure *t* = −2.60, *p* = 0.013, and a significant interaction *t* = 2.36, *p* = 0.018. Tukey contrasts for dyslexics showed significant differences between active-implausible vs. passive-implausible *z* = −3.00, *p* = 0.05 and passive-plausible vs. passive-implausible *z* = 4.59, *p* < 0.001. Neither of the other comparisons were significant. Thus, the interaction with dyslexics was driven by poorer performance in the passive-implausible condition.

#### 3.1.1. Individual Differences

The bi-variate correlations between demographic variables, individual differences variables, and comprehension accuracy are presented in Table 3. Rotation span significantly correlated with comprehension in active-plausible and passive-implausible sentences, and verbal intelligence correlated with active-implausible sentences.

**Figure 3:**
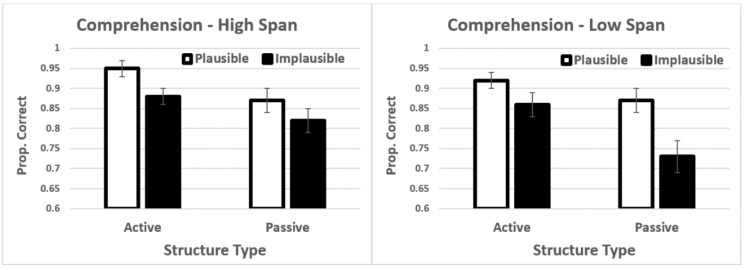
Mean comprehension accuracy by high- and low-span participants. Error bars show the standard error of the mean.

When working memory was included in the model, there was a significant main effect of sentence structure *t* = −2.37, *p* = 0.018, and a significant interaction between sentence structure and plausibility *t* = 2.75, *p* = 0.006. There was also a marginal three-way interaction between sentence structure, plausibility, and working memory *t* = −1.88, *p* = 0.06. The three-way interaction with group was not significant in this analysis (i.e., when working memory was in the model). The correlations in Table 3 show that working memory correlations with accuracy were positive. In order to conceptualise the marginal three-way interaction, we divided the sample into high-spans and low-spans. The means for each group are presented in Figure 3. As can be seen in Figure 3, the difference between high- and low-spans is quite striking. Results for the high-spans showed a double main effect: structure type and plausibility. In contrast, for low-spans, there was an interaction (compare to Figure 1). What these results showed is that participants with lower working memory showed particular difficulties with the passive-implausible sentences, similar to dyslexics in the prior analysis. However, dyslexia was not significant in this analysis. This demonstrated that there is related and overlapping variance between dyslexia status (group) and working memory, despite non-significant differences at the group level. We take this issue up in greater detail in the Discussion section. 

When verbal intelligence was included in the model, there was a significant main effect of sentence structure *t* = −2.89, *p* = 0.005, a significant main effect of verbal intelligence *t* = 2.81, *p* = 0.005, a significant interaction between sentence structure and plausibility *t* = 2.34, *p* = 0.019, and a significant three-way interaction between sentence structure, plausibility, and group *t* = −2.03, *p* = 0.043. Thus, this analysis showed that verbal intelligence exerted a main effect but did not interact with any of the other variables. The main effect was such that higher ability individuals had higher comprehension. Importantly, the three-way interaction with group was preserved even with verbal intelligence included. 

### 3.2. Eye Movements

In the analysis below, we report the total sentence reading time, and we used the same inferential statistical procedures as we did for comprehension. In order to control for the length differences in the sentences, we subtracted the total reading time on “was” and “by” (i.e., the extra words in the passives as compared with the actives). The mean reading time on “was” was 504 ms and the mean reading time on “by” was 409 ms. Examination of word skips revealed that “was” was not fixated in 11% of trials and “by” was skipped in 23% of trials. Thus, the total reading times are the total sum of fixation durations on all of the words in the active sentences, and the total sum of fixation durations for the passives excluding the reading times on the additional words, *was* and *by* (see Table 1). We also excluded reading times for trials with total reading times < 400 ms and >8000 ms, which resulted in the exclusion of 39 data points. We also provided the reading times without the adjustment for “was” and “by” in the Supplementary Materials, Section D.

#### 3.2.1. Total Reading Times

**Figure 4:**
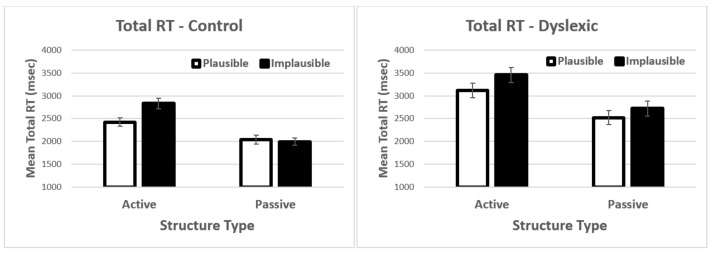
Mean total reading times. Error bars show the standard error of the mean.

Results showed significant main effects of structure type *t* = −5.52, *p* < 0.001, plausibility *t* = −3.63, *p* < 0.001, and group *t* = −3.34, *p* = 0.001 (see Figure 4). Active sentences, implausible sentences, and dyslexic participants all showed longer total reading times (see Section E, Supplementary Materials). None of the interactions were significant. Recall that the reading times on the two additional words in the passives were removed from these analyses. This is the reason for the counter-intuitive result of actives having longer reading times than passives. The unadjusted reading times are presented in the Supplementary Materials. For both analyses, the results were similar (i.e., significant main effects and no interactions). 

#### 3.2.2. Individual Differences

The bi-variate correlations between demographic variables, individual differences variables, and reading times are presented in Table 4. Rotation span significantly correlated with reading times in active-plausible and active-implausible sentences, and here, both were negative (i.e., higher span participants had lower reading times). In contrast, verbal intelligence did not correlate with reading times.

When working memory was included in the model, we observed significant main effects of structure type *t* = −3.35, *p* < 0.001 and working memory *t* = −2.71, *p* = 0.007. The main effect of group was also significant *t* = −2.44, *p* = 0.016. Again, we observed some change in the group variable with the inclusion of working memory, suggesting some overlapping variance. None of the interactions were significant.

**Figure 5:**
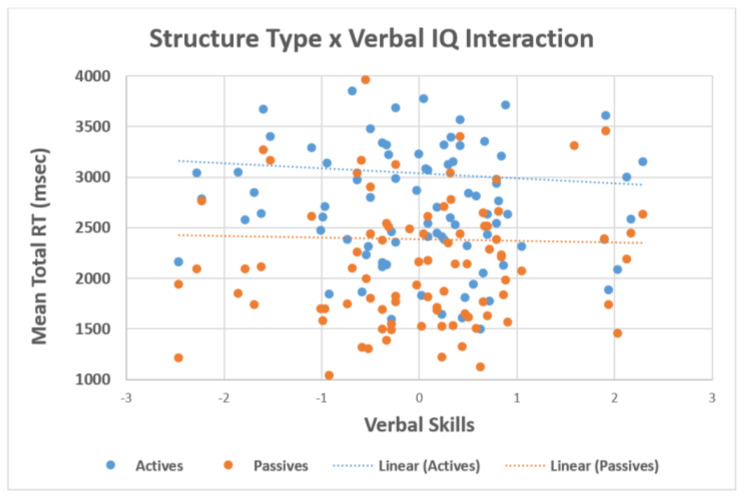
Interaction between structure type and verbal intelligence.

When verbal intelligence was included in the model, we observed significant main effects of structure *t* = −6.01, *p* < 0.001, plausibility *t* = −3.59, *p* < 0.001, and group *t* = −3.73, *p* < 0.001. In addition, there was also an interaction between structure type × verbal intelligence *t* = 2.36, *p* = 0.018, in which individuals with lower verbal intelligence had higher reading times and individuals with higher verbal intelligence had lower reading times. The effect was greater for the actives than the passives (see Figure 5). The correlations in Table 4 further highlight that the relationships were negative (i.e., individuals with higher working memory and higher verbal intelligence had lower reading times).

#### 3.2.3. Regressions per Sentence

**Figure 6:**
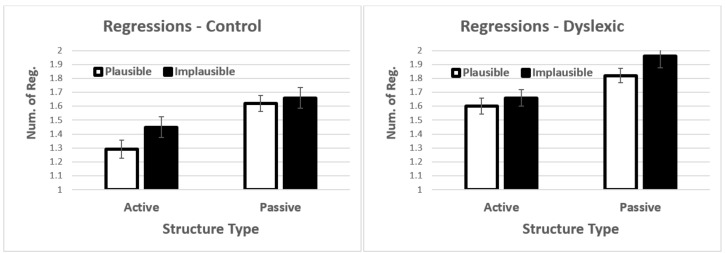
Mean number of regressions per sentence. Error bars show the standard error of the mean.

Results showed significant main effects structure type *t* = 4.64, *p* < 0.001 and group *t* = −2.23, *p* = 0.027 (see Figure 6). Passive sentences and dyslexic participants showed higher rates of regressions (see Section F, Supplementary Materials). None of the interactions were significant. 

#### 3.2.4. Individual Differences

**Figure 7:**
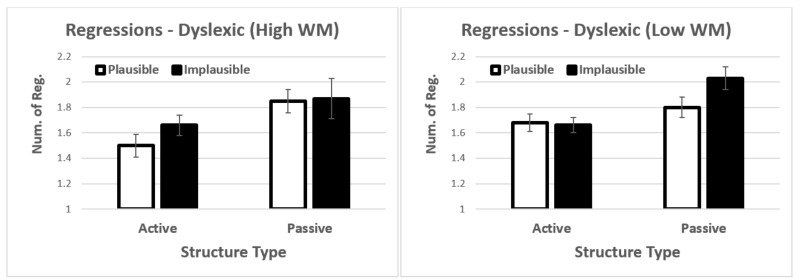
Interaction between structure type, plausibility, and working memory. Error bars show the standard error of the mean.

When working memory was included in the model, we observed a significant main effect of structure type *t* = 2.60, *p* = 0.009. In addition, there was also a significant four-way interaction *t* = −2.15, *p* = 0.032. Follow up three-way analyses showed no significant main effects or interactions in controls. For dyslexics, the three-way interaction was marginally significant *t* = 1.79, *p* = 0.075 (see Figure 7). The driving factor behind the marginal interaction was that low-span participants showed increased regressions in both the active-plausible condition and the passive-implausible conditions (i.e., the easiest and most difficult conditions). We did not explore this interaction further given that it was only marginally significant. 

When verbal intelligence was included in the model, we observed significant main effects of structure *t* = 4.42, *p* < 0.001 and group *t* = −2.11, *p* = 0.036. This is the same pattern, as when verbal intelligence was not in the model. Thus, verbal intelligence has no effect on the tendency to regress when reading. 

## 4. Discussion

In this study, we examined how dyslexic and non-dyslexic adults comprehend and process passive sentences, and we also manipulated the semantic plausibility of both active and passive sentences. Our main research objective was to investigate whether individuals with dyslexic rely more on parsing heuristics compared with non-dyslexic readers. In the Introduction, we identified several reasons why the parsing heuristics, assumed by the good-enough approach to language comprehension, would be employed more frequently in individuals with reading difficulties [11,15,27]. Our second research objective focused on the role of individual differences in two key variables (i.e., working memory and verbal intelligence). We found clear evidence that these variables affected both comprehension accuracy and reading times. However, when working memory was included in the model, the main effect of group (dyslexic vs. control) was no longer significant, which suggests that the individual differences in working memory account for shared (or overlapping) variance with dyslexia status. In contrast, with verbal intelligence in the model, the main effect of dyslexia status remained unchanged. In the remainder of the Discussion, we cover the comprehension results and reading times, as well as the implications our results have for the good-enough approach to language comprehension. 

### 4.1. Comprehension Accuracy

In terms of comprehension, we found a pattern of results that was consistent with both noun-verb-noun and semantic plausibility heuristics impacting comprehension. For controls, our results were largely consistent with the predicted pattern shown in the lower-left panel of Figure 1. For dyslexics, we found an interaction, which is consistent with the pattern shown in the lower-right panel of Figure 1. These results show similarities with Wiseheart et al.’s study [27], as they also found the same difference in comprehension between the two groups, with dyslexics showing poorer comprehension than non-dyslexics, especially with passive sentences. It is important to note that Wiseheart et al.’s [27] study did not manipulate plausibility, so this is not a direct comparison between the two experiments. 

Recall that our main research aim was to determine whether individuals with dyslexia rely on parsing heuristics to a greater extent than do controls. The interaction in our data would suggest that the answer to that is tentatively “yes”. However, there are few additional points that need to be highlighted, particularly with respect to the individual differences variables. These additional points begin to flesh out a more nuanced understanding of parsing heuristics and comprehension errors. 

Recall that our controls and dyslexics were surprisingly well matched on working memory and verbal intelligence, despite what is commonly reported in individuals with dyslexia (see Table 2). With respect to individual differences, we found that verbal intelligence resulted in a main effect, and the three-way interaction of structure, plausibility, and group was unaffected by verbal intelligence. In contrast, working memory interacted with both structure type and plausibility. In addition, the three-way interaction with dyslexia was no longer significant. The pattern of results for low-span individuals was clearly consistent with a noun-verb-noun and semantic plausibility interaction (i.e., the passive-implausible condition showed significantly lower accuracy). This finding is intriguing because it suggests that individuals with weaker working memory abilities have a greater tendency to consult real-world knowledge, and hence make a greater number of comprehension errors in sentences that are both passive and semantically implausible. Critically, this places the use of (or employment of) parsing heuristics with low-ability individuals. The second implication of these findings is that there is some relationship between working memory and dyslexia, such that when working memory in included in the model, the dyslexia effect disappears or otherwise reduces. This shows overlapping variance, and in this case, that working memory accounted for slightly more variance with the within-subjects variables than dyslexia. Plus, this is despite the groups not being significantly different in working memory. This same relationship did not exist with verbal intelligence (i.e., there does not seem to be a relationship between dyslexia and verbal intelligence). Finally, the inclusion of working memory and verbal intelligence resulted in a significant structure by plausibility interaction. This is consistent with the pattern observed in individuals with dyslexia. That interaction tends to emerge more strongly when variance is removed from the error term and built into the model (i.e., when individual differences are accounted for). Thus, our conclusion is that both noun-verb-noun and semantic plausibility heuristics are in play with these sentences and that they, in fact, interact with one another. 

### 4.2. Reading Times

The main finding with regard to reading times was that participants showed longer reading times for active sentences and dyslexics also showed longer reading times than the controls. When working memory was entered into the model, the main effect of structure remained significant, the main effect of group disappeared or reduced, and working memory was significant. This is similar to what happened with comprehension. This again suggests some degree of overlapping variance between dyslexia status and working memory, and that working memory accounts for slightly more variance than did the group. When verbal intelligence was entered into the model, plausibility was significant and there was a structure by verbal intelligence interaction. In this analysis, group also remained significant. 

There are a few points to raise with respect to reading times. First, and foremost, we removed the reading times on the two additional words to ensure that the reading times were comparable (i.e., length controlled). The unadjusted reading times are presented in the Supplementary Materials. For both analyses, there were significant main effects and no interactions. The main effect of structure (actives > passives) was obviously counter-intuitive and was the exact opposite (passives > actives) without the length adjustment. We do not view this a particularly problematic or informative. The main effect of dyslexia is important and is consistent with virtually all studies of dyslexia. We also included an additional analysis of “regressions out” in the results section. The results for regressions showed main effects of structure and group. Passive sentences and dyslexic participants showed significantly more regressions. Increased regressions in dyslexics has been reported in some prior studies but not consistently. The regression findings showed clear evidence of increased re-reading in dyslexics, and is typically inferred as an indicator of reading difficulties. Passive sentences are more complex, and so it is also expected that they show increased regressions, consistent with our data. 

### 4.3. Parsing Heuristics and Good Enough Comprehension

**Figure 8:**
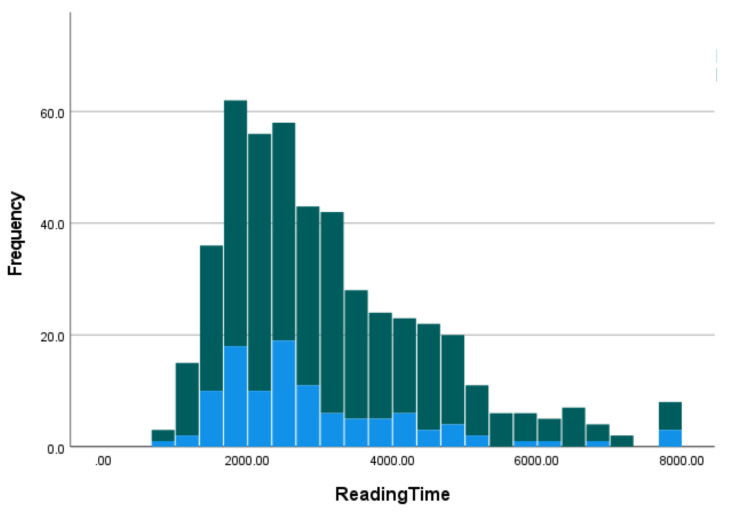
Stacked histogram showing reading times for correct trials (green) and incorrect trials (blue).

The Good Enough theory postulates the application of parsing heuristics in situations where depth of processing is not required or in cases where comprehenders seek to curtail processing effort. The latter assumption suggests positive relationships between comprehension accuracy and reading times (i.e., higher reading times would be associated with more algorithmic parsing, and lower reading times associated with strategy use). Another way to conceptualise these predictions is that one would expect the reading time distributions to be partially distinct when comparing correct and incorrect trials. In a stacked histogram this should show as a bi-modal distribution. Our data were simply not consistent with that (see Figure 8). In the Supplementary Materials (Section G, Tables S1–S3), we provided several sets of correlations, which show the relationships between reading times and comprehension accuracy. It is also important to bear in mind that only three of the four within-subject conditions were expected to show evidence of strategy use (i.e., active-implausible, passive-plausible, and passive-implausible). From the results in the Supplementary Materials, it is clear that there was very little relationship between reading times and accuracy, contrary to the assumptions of the Good Enough theory. Ultimately, there is no objective way to ascertain whether participants responses were based on heuristics (what Ferreira referred to as a pseudo-parse) or some kind of failure or error associated with the outcome of the full algorithmic parse. We have to assume that some number of the comprehension errors were due to both possibilities. However, our data clearly indicated that reading times cannot be used to differentiate these two possible sources of comprehension errors.

Interestingly, we did find that individual difference variables were significantly related to comprehension accuracy, and, specifically, verbal intelligence produced a main effect. Working memory, in contrast, interacted with both within subject variables and the pattern suggested that low-span individuals were much more likely to misinterpret passive-implausible sentences, which invites the inference that in cases where the participant has limited working memory capacity, they will tend to rely on the plausibility of events in the real-world to guide their decision making. We think that these individual differences findings open the door for a large range of new and exciting research questions concerning the use of parsing heuristics, and how and when people engage in good-enough comprehension. We suspect that some of the effect with low-span participants was made evident by the inclusion of the additional maths problem between the sentence and the comprehension question. It remains to future work to determine whether the effect of working memory on the comprehension of passive-implausible sentences is replicated without the intervening maths problem, or whether the question itself may produce some bias in participant responses. To address this second issue, a comprehension task utilising paraphrasing may be informative [60].

### 4.4. Themes of the Special Issue

We believe that there are two findings in this study that fit very well with the theme of the special issue “Expecting the Unexpected”. The first unexpected has to do with the fact that dyslexia (when included in statistical models) produced mainly main effects. The exception to this was in terms of comprehension. The strong main effects with dyslexia have also been observed in several of our past studies. In short, powerful and reliable psycholinguistic manipulations (often) do not differentially impact individuals with dyslexia, a cognitive impenetrability, if you will. The second unexpected is that the Good Enough theory makes elegant, simple, and intuitive predictions about the use of processing heuristics. It also fits well within larger research programmes and ideas (e.g., Type 1 vs. Type 2 processing). However, the behavioural data in this study did not show clear evidence for those heuristics. That is, we did not observe two clusters of results (positive correlations between processing speed and accuracy) that would be consistent with algorithmic vs. heuristic parsing. Instead, we observed essentially no correlations between accuracy and reading times. However, our individual difference findings lead to some new ways of conceptualising the Good Enough theory. Recall, according to the Good Enough theory, certain task effects (either a task is very difficult or tasks in which deep processing is not required) tend to result in heuristic parsing (and are consistent with cognitive resource conservation). This study showed that within-individual cognitive constraints (i.e., low-ability individuals) also tend to show results consistent with heuristic parsing. Whether these cases are actually heuristics or failed algorithmic parsing remains to be determined. However, this very much opens a debate about heuristic use in language processing, namely, whether it is a strategic “good” thing (used by high ability individuals) or more borne out of necessity because of cognitive limitations (in low ability individuals), as some theories explicitly postulate [61,62].

### 4.5. Strengths and Limitations

We think the main strength of this study is the large nature of the sample and the test battery. The use of a variety of cognitive assessments, as well as the fact that we tracked the eye movements of 100 participants, makes this study a rare case of a large-scale individual differences dataset from a clinical population. There were also some limitations. The first is that we had a modest number of critical items (20 in total—five per condition). The second is that our sample consisted mainly of university students, and the fact that many individuals with dyslexia do not continue into higher education means that a community-recruited dyslexia sample may show even greater differences than the ones reported here. This would particularly be the case of a sample of dyslexic individuals with lower working memory and lower verbal intelligence. The third is in regards to the assessment of working memory. We used only a single measure (rotation span), but this particular task does not include any reading or lexical components, which avoids any difficulties that dyslexic participants might have with lexical processing. In future, we would recommend using both verbal and non-verbal working memory tasks, and it is always better to have multiple measures to avoid task impurity issues. We also utilised “yes or no” (forced-choice) comprehension questions, which potentially introduced a non-canonical structure and implausibility (e.g., “did the man bite the dog?”). This could have had an effect on comprehension accuracy and the participants’ interpretation of the sentences. In future research, we would suggest the use of a paraphrasing task where participants would be required to paraphrase the sentences they have read.

## 5. Conclusions

This study aimed to investigate the processing and comprehension of passive sentences and the use of parsing heuristics in individuals with dyslexia. We also examined individual differences in verbal intelligence and working memory, their role in parsing heuristics, and their links to comprehension and reading times. Our results showed that dyslexic readers made more comprehension errors compared with controls, specifically with passive and implausible sentences. With respect to the use of parsing heuristics, our findings indicated that dyslexics show the interaction effect between noun-verb-noun and semantic plausibility. Furthermore, we found that individual differences in verbal intelligence and working memory affected both comprehension accuracy and reading times, and they seemed to be related to the use of parsing heuristics. Working memory interacted with both structure type and plausibility, which highlighted that participants with lower working memory also made more comprehension errors with passive-implausible sentences. Thus, this study implicated that parsing heuristics may be linked with the curtailment of cognitive resources in cases where cognitive resources are scarce [61,62]. The current study provides a better understanding of how dyslexic readers process and comprehend passive sentences, as well as evidence for the relationship between individual differences and the use of parsing strategies to interpret non-canonical sentences. 

## Figures and Tables

**Table 1:** Example stimuli showing passive and active and plausible and implausible sentences, and the associated comprehension questions.

Actives	
1. The dog bit the man. (Plausible)	Did the man bite the dog?
2. The man bit the dog. (Implausible)	Did the dog bite the man?
**Passives**	
3. The man was bitten by the dog. (Plausible)	Did the man bite the dog?
4. The dog was bitten by the man. (Implausible)	Did the dog bite the man?

**Table 2:** Means and standard deviations for demographic variables, Rapid Automatised Naming, verbal intelligence, and working memory for the two diagnostic groups.

	Controls (*n* = 50)	Dyslexia (*n* = 50)	*t*-Value
Variable	Mean (SD)	Mean (SD)	
Age (years)	20.31 (1.22)	21.7 (2.67)	*t* (98) = 3.34 ***
Gender (% men)	8	34	*t* (98) = 3.33 ***
RAN Letters (seconds)	12.46 (2.59)	16.50 (6.20)	*t* (98) = 4.25 ***
RAN Numbers (seconds)	11.44 (2.43)	15.26 (5.29)	*t* (98) = 4.64 ***
Similarities	93.5 (8.65)	98.8 (11.76)	*t* (98) = −2.57 *
Vocabulary	99.9 (9.18)	101.3 (9.02)	*t* (98) = −0.77
Comprehension	93.5 (10.70)	94.3 (9.31)	*t* (98) = 0.40
Verbal IQ (latent)	0.152 (0.98)	0.152 (1.00)	*t* (98) = −1.53
Rotation Span	17.7 (7.23)	16.9 (8.04)	*t* (98) = 0.51

Note. * *p* < 0.05, *** *p* < 0.001. Reported scores for RAN tasks and Rotation span are raw scores. Standard scores are reported for all other tasks.

**Table 3:** Bivariate correlations between demographics, working memory, verbal skills, and comprehension.

Variable	1	2	3	4	5	6	7	8	9
1. Age	-	0.35 **	0.32 **	0.16	0.04	0.19 #	−0.06	0.06	0.02
2. Gender		-	0.32 **	0.13	0.30 **	0.00	0.05	0.00	0.11
3. Dyslexia Status			-	−0.05	0.15	−0.29 **	−0.18 ^#^	−0.07	−0.20 #
4. Rotation Span				-	−0.04	0.26 **	0.10	0.00	0.20 *
5. Verbal Skills					-	0.03	0.27 **	0.10	0.13
6. Active-plausible						-	0.37 **	0.33 **	0.45 **
7. Active-implausible							-	0.26 **	0.34 **
8. Passive-plausible								-	0.39 **
9. Passive-implausible									-

Note. ^#^ *p* < 0.08, * *p* < 0.05, ** *p* < 0.01. Gender: 0 = woman, 1 = man; Dyslexia: 1 = dyslexic, 0 = control.

**Table 4:** Bivariate correlations between demographics, working memory, verbal skills, and total reading time on critical sentences.

Variable	1	2	3	4	5	6	7	8	9
1. Age	-	0.35 **	0.32 **	0.16	0.04	−0.07	−0.07	0.00	−0.02
2. Gender		-	0.32 **	0.13	0.30 **	0.11	0.05	0.14	0.17
3. Dyslexia Status			-	−0.05	0.15	0.33 **	0.26 *	0.27 **	0.31 **
4. Rotation Span				-	−0.04	−0.33 **	−0.27 **	−0.10	−0.19
5. Verbal Skills					-	−0.08	−0.12	−0.02	0.08
6. Active-plausible						-	0.72 **	0.67 **	0.73 **
7. Active-implausible							-	0.60 **	0.69 **
8. Passive-plausible								-	0.73 **
9. Passive-implausible									-

Note. * *p* < 0.05, ** *p* < 0.01. Gender: 0 = woman, 1 = man; Dyslexia: 1 = dyslexic, 0 = control.

## Data Availability

The data presented in this study are available on request from the corresponding author.

## Supplementary Materials

The following are available online at https://www.mdpi.com/article/10.3390/brainsci12020209/s1, Table S1: Bivariate correlations between comprehension accuracy and reading time. Table S2: Bivariate correlations between comprehension accuracy and regressions. Table S3: Mean reading times (msec) and regressions for correct and incorrect responses by group and experimental condition.
