# Supplementary material for: Use of Parsing Heuristics in the Comprehension of Passive Sentences: Evidence from Dyslexia and Individual Differences
Source: Brain Sci. 2022 Feb 1;12(2):209. doi: 10.3390/brainsci12020209 (PMC8869777; doi:10.3390/brainsci12020209)
Supplement: Supplementary file 1 [file brainsci-12-00209-s001.zip › brainsci-1535572-supplementary.pdf]

Supplementary Materials

Note that for all of the following analyses, con1 is structure (active vs. passive), con2 is plausibility (plausible vs. implausible), and dys is group (dyslexic vs. control).

Section A: R output for the analysis of length and Frequency on length adjusted reading times. Results showed a main effect of length, despite the length correction for the additional words in passive sentences. The inclusion of word frequency (summed frequency of the three content words) did not produce a main effect.

```
Linear mixed model fit by maximum likelihood . t-tests use Satterthwaite's method ['lmerModLmerTest']
Formula: rtlen ~ con1 * con2 * dys + len + (1 + con1 | sub) + (1 | item)
   Data: paul

     AIC      BIC   logLik deviance df.resid
 34415.2  34493.6 -17193.6  34387.2     1986

Scaled residuals:
    Min      1Q  Median      3Q     Max
-2.6061 -0.5901 -0.1421  0.3940  8.1369

Random effects:
 Groups   Name        Variance Std.Dev. Corr
 sub      (Intercept)  834115   913.3
          con1c2       166408   407.9   -0.33
 item     (Intercept)    6305    79.4
 Residual             1483300  1217.9
Number of obs: 2000, groups:  sub, 100; item, 20

Fixed effects:
                    Estimate Std. Error       df t value Pr(>|t|)
(Intercept)         2708.665    196.834   74.609  13.761  < 2e-16 ***
con1c2              -920.808    130.140  140.189  -7.076 6.51e-11 ***
con2c2              -381.612    108.933 1780.543  -3.503 0.000471 ***
dysd2               -609.696    212.676  132.308  -2.867 0.004826 **
len                   47.514      7.073   19.149   6.717 1.94e-06 ***
con1c2:con2c2        222.548    154.055 1780.543   1.445 0.148745
con1c2:dysd2         -74.004    174.325  263.026  -0.425 0.671535
con2c2:dysd2         -78.748    154.055 1780.543  -0.511 0.609296
con1c2:con2c2:dysd2  193.024    217.866 1780.543   0.886 0.375750
---
Signif. codes:  0 '***' 0.001 '**' 0.01 '*' 0.05 '.' 0.1 ' ' 1

Correlation of Fixed Effects:
            (Intr) con1c2 con2c2 dysd2  len    cn12:22 cn12:2 cn22:2
con1c2      -0.246
con2c2      -0.277  0.419
dysd2       -0.540  0.303  0.256
len         -0.632 -0.168  0.000  0.000
cn1c2:cn2c2  0.196 -0.592 -0.707 -0.181  0.000
cn1c2:dysd2  0.245 -0.670 -0.312 -0.453  0.000  0.442
cn2c2:dysd2  0.196 -0.296 -0.707 -0.362  0.000  0.500   0.442
cn1c2:c22:2 -0.138  0.419  0.500  0.256  0.000 -0.707  -0.625 -0.707
```

```
Linear mixed model fit by maximum likelihood . t-tests use Satterthwaite's method ['lmerModLmerTest']
Formula: rtlen ~ con1 * con2 * dys + fre + (1 + con1 | sub) + (1 | item)
   Data: paul

     AIC      BIC   logLik deviance df.resid
 34436.8  34515.2 -17204.4  34408.8     1986

Scaled residuals:
    Min      1Q  Median      3Q     Max
-2.5589 -0.5859 -0.1361  0.3832  8.1276

Random effects:
 Groups   Name        Variance Std.Dev. Corr
 sub      (Intercept)  836220   914.5
          con1c2       168716   410.8   -0.33
 item     (Intercept)   49762   223.1
 Residual             1483033  1217.8
Number of obs: 2000, groups:  sub, 100; item, 20

Fixed effects:
                     Estimate Std. Error        df t value Pr(>|t|)
(Intercept)         3582.0973   168.5962  122.1969  21.247  < 2e-16 ***
con1c2              -692.8933   170.0353   55.2812  -4.075 0.000148 ***
con2c2              -381.6120   108.9232 1781.4222  -3.503 0.000471 ***
dysd2               -609.6960   212.8687  132.1545  -2.864 0.004865 **
fre                   -0.4454     0.3370   19.4960  -1.322 0.201571
con1c2:con2c2        222.5480   154.0407 1781.4222   1.445 0.148708
con1c2:dysd2         -74.0040   174.5771  260.9358  -0.424 0.671985
con2c2:dysd2         -78.7480   154.0407 1781.4222  -0.511 0.609263
con1c2:con2c2:dysd2  193.0240   217.8464 1781.4222   0.886 0.375707
---
Signif. codes:  0 '***' 0.001 '**' 0.01 '*' 0.05 '.' 0.1 ' ' 1

Correlation of Fixed Effects:
            (Intr) con1c2 con2c2 dysd2  fre    cn12:22 cn12:2 cn22:2
con1c2      -0.407
con2c2      -0.323  0.320
dysd2       -0.631  0.232  0.256
fre         -0.167 -0.359  0.000  0.000
cn1c2:cn2c2  0.228 -0.453 -0.707 -0.181  0.000
cn1c2:dysd2  0.286 -0.513 -0.312 -0.453  0.000  0.441
cn2c2:dysd2  0.228 -0.226 -0.707 -0.362  0.000  0.500   0.441
cn1c2:c22:2 -0.162  0.320  0.500  0.256  0.000 -0.707  -0.624 -0.707
```

Section B: R output for the analysis of comprehension accuracy. First output is the three-way interaction. Second and third are the results for dyslexics and controls separately. The final output is the Tukey contrasts.

```
Linear mixed model fit by maximum likelihood . t-tests use Satterthwaite's
Formula: acc ~ con1 * con2 * dys + (1 + con1 | sub) + (1 | item)
   Data: paul

     AIC      BIC   logLik deviance df.resid
  1359.6   1432.4   -666.8   1333.6     1987

Scaled residuals:
     Min       1Q   Median       3Q      Max
-3.09493  0.01579  0.26466  0.50720  1.92154

Random effects:
 Groups   Name        Variance Std.Dev. Corr
 sub      (Intercept) 0.015571 0.12478
          con1c2      0.007469 0.08642  -0.10
 item     (Intercept) 0.002106 0.04589
 Residual             0.103542 0.32178
Number of obs: 2000, groups:  sub, 100; item, 20

Fixed effects:
                      Estimate Std. Error         df t value Pr(>|t|)
(Intercept)            0.83600    0.03060  112.97594  27.323  < 2e-16 ***
con1c2                -0.11200    0.03740   83.81125  -2.995  0.00361 **
con2c2                 0.03200    0.02878 1780.96958   1.112  0.26635
dysd2                  0.03600    0.03809  193.73696   0.945  0.34583
con1c2:con2c2          0.10000    0.04070 1780.96958   2.457  0.01411 *
con1c2:dysd2           0.06400    0.04422  291.14032   1.447  0.14889
con2c2:dysd2           0.04000    0.04070 1780.96958   0.983  0.32586
con1c2:con2c2:dysd2   -0.11600    0.05756 1780.96958  -2.015  0.04403 *
---
Signif. codes:  0 '***' 0.001 '**' 0.01 '*' 0.05 '.' 0.1 ' ' 1

Correlation of Fixed Effects:
            (Intr) con1c2 con2c2 dysd2  cn12:22 cn12:2 cn22:2
con1c2      -0.565
con2c2      -0.470  0.385
dysd2       -0.623  0.306  0.378
cn1c2:cn2c2  0.333 -0.544 -0.707 -0.267
cn1c2:dysd2  0.322 -0.591 -0.325 -0.518  0.460
cn2c2:dysd2  0.333 -0.272 -0.707 -0.534  0.500   0.460
cn1c2:c22:2 -0.235  0.385  0.500  0.378 -0.707  -0.651 -0.707
```

Controls

```
Linear mixed model fit by maximum likelihood . t-tests use Satterthwaite's method ['lmerModLmerTest']
Formula: acc ~ con1 * con2 + (1 | sub) + (1 | item)
   Data: paul

     AIC      BIC   logLik deviance df.resid 
   562.3    596.7   -274.2    548.3      993 

Scaled residuals: 
    Min      1Q  Median      3Q     Max 
-3.1208  0.1216  0.3090  0.4584  1.2618 

Random effects:
 Groups   Name        Variance  Std.Dev.
 sub      (Intercept) 0.0067083 0.08190 
 item     (Intercept) 0.0006736 0.02595 
 Residual             0.0964285 0.31053 
Number of obs: 1000, groups:  sub, 50; item, 20

Fixed effects:
               Estimate Std. Error        df t value Pr(>|t|)    
(Intercept)     0.87200    0.02423  75.69144  35.984  < 2e-16 ***
con1c2         -0.04800    0.03010  59.16608  -1.595  0.11614    
con2c2          0.07200    0.02777 930.13631   2.592  0.00968 ** 
con1c2:con2c2  -0.01600    0.03928 930.13631  -0.407  0.68385    
---
Signif. codes:  0 '***' 0.001 '**' 0.01 '*' 0.05 '.' 0.1 ' ' 1

Correlation of Fixed Effects:
            (Intr) con1c2 con2c2
con1c2      -0.621              
con2c2      -0.573  0.461       
cn1c2:cn2c2  0.405 -0.652 -0.707
> 
```

Dyslexia

```
Linear mixed model fit by maximum likelihood . t-tests use Satterthwaite's method ['lmerModLmerTest']
Formula: acc ~ con1 * con2 + (1 + con1 | sub) + (1 | item)
   Data: paul

     AIC      BIC   logLik deviance df.resid 
   787.6    831.8   -384.8    769.6      991 

Scaled residuals: 
     Min       1Q   Median       3Q      Max 
-2.88255 -0.03895  0.24272  0.56420  1.84661 

Random effects:
 Groups   Name        Variance Std.Dev. Corr 
 sub      (Intercept) 0.024722 0.15723       
          con1c2      0.007891 0.08883  -0.05
 item     (Intercept) 0.004006 0.06329       
 Residual             0.112048 0.33474       
Number of obs: 1000, groups:  sub, 50; item, 20

Fixed effects:
               Estimate Std. Error        df t value Pr(>|t|)    
(Intercept)     0.83600    0.03665  59.05789  22.810   <2e-16 ***
con1c2         -0.11200    0.04307  39.26942  -2.600   0.0131 *  
con2c2          0.03200    0.02994 880.96805   1.069   0.2854    
con1c2:con2c2   0.10000    0.04234 880.96805   2.362   0.0184 *  
---
Signif. codes:  0 '***' 0.001 '**' 0.01 '*' 0.05 '.' 0.1 ' ' 1

Correlation of Fixed Effects:
            (Intr) con1c2 con2c2
con1c2      -0.547              
con2c2      -0.408  0.348       
cn1c2:cn2c2  0.289 -0.491 -0.707
```

Tukey Contrasts: first variable is structure type (C1=active, C2=passive), second is plausibility (C1=implausible, C2=plausible, and third is group (D1=dyslexic, D2=Control).

```
         Simultaneous Tests for General Linear Hypotheses

Multiple Comparisons of Means: Tukey Contrasts


Fit: lmer(formula = acc ~ tw + (1 + con1 | sub) + (1 | item), data = paul,
    REML = FALSE)

Linear Hypotheses:
                         Estimate Std. Error z value Pr(>|z|)
c2.c1.d1 - c1.c1.d1 == 0 -0.11200    0.03740  -2.995  0.05038 .
c1.c2.d1 - c1.c1.d1 == 0  0.03200    0.02878   1.112  0.94700
c2.c2.d1 - c1.c1.d1 == 0  0.02000    0.03740   0.535  0.99935
c1.c1.d2 - c1.c1.d1 == 0  0.03600    0.03809   0.945  0.97810
c2.c1.d2 - c1.c1.d1 == 0 -0.01200    0.04448  -0.270  0.99999
c1.c2.d2 - c1.c1.d1 == 0  0.10800    0.03809   2.835  0.07883 .
c2.c2.d2 - c1.c1.d1 == 0  0.04400    0.04448   0.989  0.97170
c1.c2.d1 - c2.c1.d1 == 0  0.14400    0.03740   3.850  0.00269 **
c2.c2.d1 - c2.c1.d1 == 0  0.13200    0.02878   4.586  < 0.001 ***
c1.c1.d2 - c2.c1.d1 == 0  0.14800    0.04448   3.328  0.01810 *
c2.c1.d2 - c2.c1.d1 == 0  0.10000    0.04078   2.452  0.19878
c1.c2.d2 - c2.c1.d1 == 0  0.22000    0.04448   4.946  < 0.001 ***
c2.c2.d2 - c2.c1.d1 == 0  0.15600    0.04078   3.826  0.00299 **
c2.c2.d1 - c1.c2.d1 == 0 -0.01200    0.03740  -0.321  0.99998
c1.c1.d2 - c1.c2.d1 == 0  0.00400    0.03809   0.105  1.00000
c2.c1.d2 - c1.c2.d1 == 0 -0.04400    0.04448  -0.989  0.97170
c1.c2.d2 - c1.c2.d1 == 0  0.07600    0.03809   1.995  0.45691
c2.c2.d2 - c1.c2.d1 == 0  0.01200    0.04448   0.270  0.99999
c1.c1.d2 - c2.c2.d1 == 0  0.01600    0.04448   0.360  0.99995
c2.c1.d2 - c2.c2.d1 == 0 -0.03200    0.04078  -0.785  0.99265
c1.c2.d2 - c2.c2.d1 == 0  0.08800    0.04448   1.979  0.46817
c2.c2.d2 - c2.c2.d1 == 0  0.02400    0.04078   0.589  0.99879
c2.c1.d2 - c1.c1.d2 == 0 -0.04800    0.03740  -1.283  0.89191
c1.c2.d2 - c1.c1.d2 == 0  0.07200    0.02878   2.502  0.17860
c2.c2.d2 - c1.c1.d2 == 0  0.00800    0.03740   0.214  1.00000
c1.c2.d2 - c2.c1.d2 == 0  0.12000    0.03740   3.208  0.02640 *
c2.c2.d2 - c2.c1.d2 == 0  0.05600    0.02878   1.946  0.49005
c2.c2.d2 - c1.c2.d2 == 0 -0.06400    0.03740  -1.711  0.65319
---
Signif. codes:  0 '***' 0.001 '**' 0.01 '*' 0.05 '.' 0.1 ' ' 1
(Adjusted p values reported -- single-step method)
```

Section C: R output for the analysis of comprehension accuracy with the inclusion of individual differences variables. Working Memory followed by Verbal Intelligence.

```
Linear mixed model fit by maximum likelihood . t-tests use Satterthwaite's method ['lmerModLmerTest']
Formula: acc ~ con1 * con2 * dys * wm + (1 + con1 | sub) + (1 | item)
   Data: paul

     AIC      BIC   logLik deviance df.resid
  1363.5   1481.1   -660.7   1321.5     1979

Scaled residuals:
     Min       1Q   Median       3Q      Max
-3.12416  0.02797  0.26641  0.49235  1.93968

Random effects:
 Groups   Name        Variance Std.Dev. Corr
 sub      (Intercept) 0.014443 0.12018
          con1c2      0.007458 0.08636  -0.08
 item     (Intercept) 0.002093 0.04575
 Residual             0.103128 0.32114
Number of obs: 2000, groups:  sub, 100; item, 20

Fixed effects:
                        Estimate Std. Error         df t value Pr(>|t|)
(Intercept)            7.518e-01  6.399e-02  2.067e+02  11.749  < 2e-16 ***
con1c2                -1.807e-01  7.628e-02  2.818e+02  -2.370  0.01848 *
con2c2                 2.415e-02  6.770e-02  1.787e+03   0.357  0.72128
dysd2                  7.346e-02  9.422e-02  1.984e+02   0.780  0.43652
wm                     4.968e-03  3.331e-03  1.989e+02   1.491  0.13743
con1c2:con2c2          2.630e-01  9.574e-02  1.787e+03   2.747  0.00607 **
con1c2:dysd2           1.191e-01  1.110e-01  2.910e+02   1.073  0.28432
con2c2:dysd2           1.807e-02  1.022e-01  1.781e+03   0.177  0.85967
con1c2:wm              4.058e-03  3.927e-03  2.921e+02   1.033  0.30226
con2c2:wm              4.631e-04  3.619e-03  1.789e+03   0.128  0.89819
dysd2:wm              -2.333e-03  4.976e-03  1.984e+02  -0.469  0.63973
con1c2:con2c2:dysd2   -1.912e-01  1.445e-01  1.781e+03  -1.323  0.18608
con1c2:con2c2:wm      -9.624e-03  5.118e-03  1.789e+03  -1.881  0.06019 .
con1c2:dysd2:wm       -3.289e-03  5.864e-03  2.910e+02  -0.561  0.57538
con2c2:dysd2:wm        1.217e-03  5.397e-03  1.781e+03   0.226  0.82157
con1c2:con2c2:dysd2:wm 4.665e-03  7.632e-03  1.781e+03   0.611  0.54109
---
Signif. codes:  0 '***' 0.001 '**' 0.01 '*' 0.05 '.' 0.1 ' ' 1

Correlation matrix not shown by default, as p = 16 > 12.
Use print(x, correlation=TRUE)  or
    vcov(x)        if you need it
```

```
Linear mixed model fit by maximum likelihood . t-tests use Satterthwaite's method ['lmerModLmerTest']
Formula: acc ~ con1 * con2 * dys * viq + (1 + con1 | sub) + (1 | item)
   Data: paul

     AIC      BIC   logLik deviance df.resid
  1364.9   1482.5   -661.4   1322.9     1979

Scaled residuals:
     Min       1Q   Median       3Q      Max
-3.16181  0.02104  0.26288  0.50751  1.95221

Random effects:
 Groups   Name        Variance Std.Dev. Corr
 sub      (Intercept) 0.013918 0.11798
          con1c2      0.007469 0.08643  -0.09
 item     (Intercept) 0.002092 0.04574
 Residual             0.103349 0.32148
Number of obs: 2000, groups:  sub, 100; item, 20

Fixed effects:
                          Estimate Std. Error         df t value Pr(>|t|)
(Intercept)                0.82469    0.03028  112.45275  27.232  < 2e-16 ***
con1c2                    -0.10904    0.03765   86.27113  -2.896  0.00478 **
con2c2                     0.03793    0.02909 1781.10014   1.304  0.19240
dysd2                      0.04992    0.03764  200.99773   1.326  0.18626
viq                        0.07442    0.02648  201.50776   2.810  0.00543 **
con1c2:con2c2              0.09622    0.04114 1781.10014   2.339  0.01945 *
con1c2:dysd2               0.06325    0.04471  291.10789   1.415  0.15827
con2c2:dysd2               0.03475    0.04115 1781.56448   0.844  0.39863
con1c2:viq                -0.01947    0.03146  292.13080  -0.619  0.53654
con2c2:viq                -0.03905    0.02900 1788.62780  -1.347  0.17826
dysd2:viq                 -0.05723    0.03784  201.05239  -1.512  0.13202
con1c2:con2c2:dysd2       -0.11813    0.05820 1781.56448  -2.030  0.04253 *
con1c2:con2c2:viq          0.02486    0.04101 1788.62780   0.606  0.54436
con1c2:dysd2:viq           0.03400    0.04495  291.21768   0.756  0.45006
con2c2:dysd2:viq           0.04352    0.04138 1782.39564   1.052  0.29313
con1c2:con2c2:dysd2:viq   -0.06377    0.05852 1782.39564  -1.090  0.27601
---
Signif. codes:  0 '***' 0.001 '**' 0.01 '*' 0.05 '.' 0.1 ' ' 1

Correlation matrix not shown by default, as p = 16 > 12.
Use print(x, correlation=TRUE)  or
    vcov(x)        if you need it
```

Section D: R output for the analysis of un-adjusted reading times.

```
Linear mixed model fit by maximum likelihood . t-tests use Satterthwaite's method ['lmerModLmerTest']
Formula: rt ~ conl * con2 * dys + (1 + conl | sub) + (1 | item)
   Data: paul

     AIC      BIC   logLik deviance df.resid 
 33035.8  33108.2 -16504.9  33009.8     1934 

Scaled residuals: 
    Min      1Q  Median      3Q     Max 
-2.4757 -0.6430 -0.1588  0.4786  4.2753 

Random effects:
 Groups   Name        Variance Std.Dev. Corr
 sub      (Intercept)  640960   800.6       
          conlc2        66708   258.3   0.14
 item     (Intercept)   36477   191.0       
 Residual             1156577  1075.4       
Number of obs: 1947, groups:  sub, 100; item, 20

Fixed effects:
                    Estimate Std. Error      df t value Pr(>|t|)    
(Intercept)          3453.98     145.65  126.30  23.714  < 2e-16 ***
conlc2                128.40     135.36   64.54   0.949 0.346386    
con2c2               -341.06      97.21 1724.64  -3.509 0.000462 ***
dysd2                -622.21     187.33  131.68  -3.322 0.001159 ** 
conlc2:con2c2          28.93     138.73 1729.91   0.209 0.834840    
conlc2:dysd2         -177.18     147.57  294.63  -1.201 0.230831    
con2c2:dysd2          -61.15     137.26 1724.61  -0.446 0.656000    
conlc2:con2c2:dysd2   280.59     195.18 1727.96   1.438 0.150727    
---
Signif. codes:  0 '***' 0.001 '**' 0.01 '*' 0.05 '.' 0.1 ' ' 1

Correlation of Fixed Effects:
            (Intr) conlc2 con2c2 dysd2  cnl2:22 cnl2:2 cn22:2
conlc2      -0.397                                           
con2c2      -0.335  0.360                                    
dysd2       -0.644  0.165  0.261                             
cnlc2:cn2c2  0.235 -0.516 -0.700 -0.182                      
cnlc2:dysd2  0.194 -0.552 -0.331 -0.301  0.473               
cn2c2:dysd2  0.237 -0.255 -0.708 -0.368  0.496   0.466       
cnlc2:c22:2 -0.167  0.367  0.498  0.258 -0.711  -0.663 -0.703
```

Means of unadjusted total reading times.

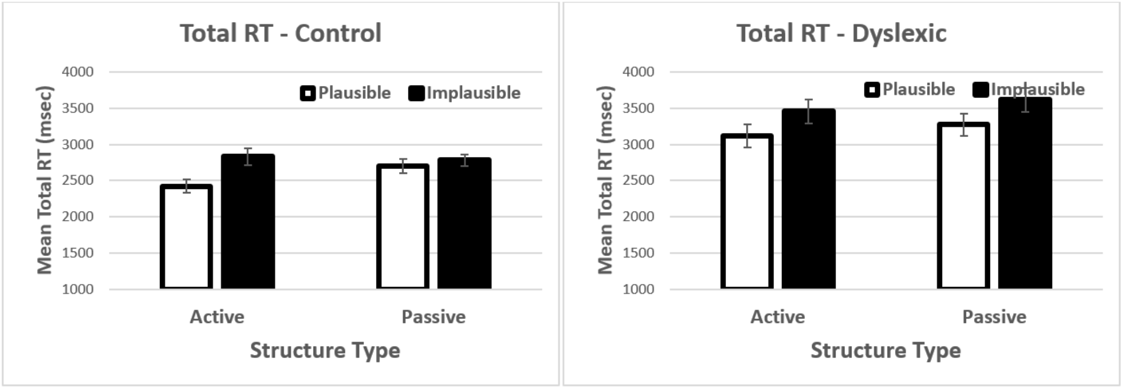

Working Memory

```
Linear mixed model fit by maximum likelihood . t-tests use Satterthwaite's method ['lmerModLmerTest']
Formula: rt ~ con1 * con2 * dys * wm + (1 + con1 | sub) + (1 | item)
   Data: paul

     AIC      BIC   logLik deviance df.resid
 33039.8  33156.8 -16498.9  32997.8     1926

Scaled residuals:
    Min      1Q  Median      3Q     Max
-2.4380 -0.6486 -0.1595  0.4688  4.3073

Random effects:
 Groups   Name        Variance Std.Dev. Corr
 sub      (Intercept)  572257   756.5
          con1c2        57724   240.3   0.26
 item     (Intercept)   36382   190.7
 Residual             1156212  1075.3
Number of obs: 1947, groups:  sub, 100; item, 20

Fixed effects:
                        Estimate Std. Error       df t value Pr(>|t|)
(Intercept)             4161.492    306.472  145.997  13.579   <2e-16 ***
con1c2                   -18.971    263.883  287.312  -0.072   0.9427
con2c2                  -202.188    231.381 1729.316  -0.874   0.3823
dysd2                  -1067.763    454.135  137.607  -2.351   0.0201 *
wm                       -41.770     16.025  136.933  -2.607   0.0102 *
con1c2:con2c2            -36.278    332.571 1741.900  -0.109   0.9131
con1c2:dysd2            -325.171    371.838  309.365  -0.874   0.3825
con2c2:dysd2            -311.327    348.576 1725.869  -0.893   0.3719
con1c2:wm                  8.637     13.229  314.868   0.653   0.5143
con2c2:wm                 -8.107     12.300 1729.613  -0.659   0.5099
dysd2:wm                  27.056     23.940  136.654   1.130   0.2604
con1c2:con2c2:dysd2      461.178    495.812 1732.091   0.930   0.3524
con1c2:con2c2:wm           3.840     17.675 1738.894   0.217   0.8280
con1c2:dysd2:wm            7.970     19.540  304.710   0.408   0.6836
con2c2:dysd2:wm           14.329     18.323 1724.981   0.782   0.4343
con1c2:con2c2:dysd2:wm   -10.324     26.082 1729.576  -0.396   0.6923
---
Signif. codes:  0 '***' 0.001 '**' 0.01 '*' 0.05 '.' 0.1 ' ' 1

Correlation matrix not shown by default, as p = 16 > 12.
Use print(x, correlation=TRUE)  or
    vcov(x)        if you need it
```

Verbal Intelligence

```
Linear mixed model fit by maximum likelihood . t-tests use Satterthwaite's method ['lmerModLmerTest']
Formula: rt ~ con1 * con2 * dys * viq + (1 + con1 | sub) + (1 | item)
   Data: paul

     AIC      BIC   logLik deviance df.resid 
 33033.0  33150.1 -16495.5  32991.0     1926 

Scaled residuals: 
    Min      1Q  Median      3Q     Max 
-2.4791 -0.6566 -0.1663  0.4881  4.2589 

Random effects:
 Groups   Name        Variance Std.Dev. Corr
 sub      (Intercept)  619718   787.2       
          con1c2        29902   172.9   0.38
 item     (Intercept)   35185   187.6       
 Residual             1154096  1074.3       
Number of obs: 1947, groups:  sub, 100; item, 20

Fixed effects:
                        Estimate Std. Error      df t value Pr(>|t|)    
(Intercept)              3479.30     145.11  127.51  23.976  < 2e-16 ***
con1c2                     77.11     132.45   63.01   0.582 0.562533    
con2c2                   -344.51      98.24 1726.19  -3.507 0.000465 ***
dysd2                    -678.23     187.24  132.71  -3.622 0.000414 ***
viq                      -164.76     131.69  132.93  -1.251 0.213090    
con1c2:con2c2              47.01     140.09 1731.22   0.336 0.737243    
con1c2:dysd2             -106.76     144.04  329.96  -0.741 0.459108    
con2c2:dysd2              -38.85     138.76 1726.71  -0.280 0.779549    
con1c2:viq                337.89     101.91  336.57   3.316 0.001014 ** 
con2c2:viq                 19.11      97.65 1731.68   0.196 0.844889    
dysd2:viq                 -33.80     188.37  133.10  -0.179 0.857885    
con1c2:con2c2:dysd2       227.58     197.22 1729.88   1.154 0.248688    
con1c2:con2c2:viq        -111.95     139.35 1734.41  -0.803 0.421895    
con1c2:dysd2:viq         -212.71     145.07  332.54  -1.466 0.143511    
con2c2:dysd2:viq          104.82     139.84 1727.22   0.750 0.453599    
con1c2:con2c2:dysd2:viq  -117.12     198.49 1729.65  -0.590 0.555208    
---
Signif. codes:  0 '***' 0.001 '**' 0.01 '*' 0.05 '.' 0.1 ' ' 1

Correlation matrix not shown by default, as p = 16 > 12.
Use print(x, correlation=TRUE)  or
    vcov(x)        if you need it
```

Section E: R output for the analysis of length adjusted reading times.

```
Linear mixed model fit by maximum likelihood . t-tests use Satterthwaite's method ['lmerModLmerTest']
Formula: rtlen ~ con1 * con2 * dys + (1 + con1 | sub) + (1 | item)
   Data: paul

     AIC      BIC   logLik deviance df.resid
 33151.1  33223.7 -16562.6  33125.1     1948

Scaled residuals:
    Min      1Q  Median      3Q     Max
-2.4459 -0.6432 -0.1371  0.4595  4.7761

Random effects:
 Groups   Name        Variance Std.Dev. Corr
 sub      (Intercept)  653203   808.2
          con1c2        67966   260.7   -0.36
 item     (Intercept)   39507   198.8
 Residual             1089022  1043.6
Number of obs: 1961, groups:  sub, 100; item, 20

Fixed effects:
                    Estimate Std. Error      df t value Pr(>|t|)
(Intercept)          3457.40     146.58  124.37  23.587  < 2e-16 ***
con1c2               -744.75     134.84   59.01  -5.523 7.87e-07 ***
con2c2               -342.51      94.33 1742.86  -3.631  0.00029 ***
dysd2                -625.23     187.16  129.46  -3.341  0.00109 **
con1c2:con2c2         150.17     133.41 1742.83   1.126  0.26045
con1c2:dysd2          -92.30     143.19  299.40  -0.645  0.51966
con2c2:dysd2          -58.31     133.19 1742.80  -0.438  0.66161
con1c2:con2c2:dysd2   280.38     188.64 1744.02   1.486  0.13738
---
Signif. codes:  0 '***' 0.001 '**' 0.01 '*' 0.05 '.' 0.1 ' ' 1

Correlation of Fixed Effects:
            (Intr) con1c2 con2c2 dysd2  cn12:22 cn12:2 cn22:2
con1c2      -0.502
con2c2      -0.323  0.351
dysd2       -0.639  0.237  0.253
cn1c2:cn2c2  0.229 -0.496 -0.707 -0.179
cn1c2:dysd2  0.285 -0.532 -0.331 -0.445  0.467
cn2c2:dysd2  0.229 -0.249 -0.708 -0.357  0.501   0.467
cn1c2:c22:2 -0.162  0.351  0.500  0.252 -0.707  -0.658 -0.706
```

Working Memory

```
Linear mixed model fit by maximum likelihood . t-tests use Satterthwaite's method ['lmerModLmerTest']
Formula: rtlen ~ con1 * con2 * dys * wm + (1 | sub) + (1 | item)
   Data: paul

     AIC      BIC   logLik deviance df.resid
 33153.6  33259.6 -16557.8  33115.6     1942

Scaled residuals:
    Min      1Q  Median      3Q     Max
-2.4581 -0.6535 -0.1428  0.4659  4.6634

Random effects:
 Groups   Name        Variance Std.Dev.
 sub      (Intercept)  545577   738.6
 item     (Intercept)   38927   197.3
 Residual             1103239  1050.4
Number of obs: 1961, groups:  sub, 100; item, 20

Fixed effects:
                        Estimate Std. Error       df t value Pr(>|t|)
(Intercept)             4174.732    299.975  174.927  13.917  < 2e-16 ***
con1c2                  -809.151    241.858  528.253  -3.346  0.00088 ***
con2c2                  -207.413    226.024 1846.154  -0.918  0.35892
dysd2                  -1082.608    443.461  164.705  -2.441  0.01569 *
wm                       -42.375     15.648  163.900  -2.708  0.00749 **
con1c2:con2c2           -253.323    318.407 1846.171  -0.796  0.42637
con1c2:dysd2            -178.084    339.502 1841.503  -0.525  0.59996
con2c2:dysd2            -299.995    340.479 1841.538  -0.881  0.37838
con1c2:wm                  3.785     12.000 1844.624   0.315  0.75247
con2c2:wm                 -7.809     12.016 1847.010  -0.650  0.51582
dysd2:wm                  27.743     23.378  163.565   1.187  0.23706
con1c2:con2c2:dysd2      738.202    479.389 1841.495   1.540  0.12376
con1c2:con2c2:wm          23.687     16.981 1847.035   1.395  0.16323
con1c2:dysd2:wm            4.639     17.882 1841.421   0.259  0.79535
con2c2:dysd2:wm           13.803     17.898 1841.357   0.771  0.44069
con1c2:con2c2:dysd2:wm   -26.767     25.276 1841.373  -1.059  0.28975
---
Signif. codes:  0 '***' 0.001 '**' 0.01 '*' 0.05 '.' 0.1 ' ' 1

Correlation matrix not shown by default, as p = 16 > 12.
Use print(x, correlation=TRUE)  or
    vcov(x)        if you need it
```

Verbal Intelligence

```
Linear mixed model fit by maximum likelihood . t-tests use Satterthwaite's method ['lmerModLmerTest']
Formula: rtlen ~ con1 * con2 * dys * viq + (1 | sub) + (1 | item)
   Data: paul

     AIC      BIC   logLik deviance df.resid
 33151.3  33257.3 -16556.6  33113.3     1942

Scaled residuals:
    Min      1Q  Median      3Q     Max
-2.5046 -0.6505 -0.1384  0.4615  4.8692

Random effects:
 Groups   Name        Variance Std.Dev.
 sub      (Intercept)  587042   766.2
 item     (Intercept)   37776   194.4
 Residual             1098188  1047.9
Number of obs: 1961, groups:  sub, 100; item, 20

Fixed effects:
                        Estimate Std. Error      df t value Pr(>|t|)
(Intercept)              3481.59     142.87  138.57  24.368  < 2e-16 ***
con1c2                   -778.34     129.45   56.60  -6.013  1.4e-07 ***
con2c2                   -344.26      95.83 1841.26  -3.592 0.000336 ***
dysd2                    -680.89     182.35  157.49  -3.734 0.000263 ***
viq                      -163.79     128.25  157.75  -1.277 0.203444
con1c2:con2c2             151.70     135.45 1841.29   1.120 0.262877
con1c2:dysd2              -28.60     135.51 1841.57  -0.211 0.832848
con2c2:dysd2              -37.01     135.35 1841.80  -0.273 0.784569
con1c2:viq                226.26      95.70 1844.37   2.364 0.018166 *
con2c2:viq                 17.98      95.27 1847.27   0.189 0.850350
dysd2:viq                 -38.08     183.45  157.95  -0.208 0.835820
con1c2:con2c2:dysd2       233.85     191.71 1841.98   1.220 0.222694
con1c2:con2c2:viq         -13.29     135.10 1846.89  -0.098 0.921648
con1c2:dysd2:viq          -29.30     136.78 1841.88  -0.214 0.830395
con2c2:dysd2:viq          105.25     136.41 1842.04   0.772 0.440457
con1c2:con2c2:dysd2:viq  -265.74     193.01 1842.29  -1.377 0.168733
---
Signif. codes:  0 '***' 0.001 '**' 0.01 '*' 0.05 '.' 0.1 ' ' 1

Correlation matrix not shown by default, as p = 16 > 12.
Use print(x, correlation=TRUE)  or
    vcov(x)        if you need it
```

Section F: R output for the analysis of regressions out.

```
Linear mixed model fit by maximum likelihood . t-tests use Satterthwaite's method ['lmerModLmerTest']
Formula: reg ~ con1 * con2 * dys + (1 | sub) + (1 | item)
   Data: paul

     AIC      BIC   logLik deviance df.resid
  4667.1   4728.7  -2322.5   4645.1     1989

Scaled residuals:
    Min      1Q  Median      3Q     Max
-2.8679 -0.7583  0.0389  0.6028  4.0445

Random effects:
 Groups   Name        Variance Std.Dev.
 sub      (Intercept) 0.1073   0.3276
 item     (Intercept) 0.0000   0.0000
 Residual             0.5517   0.7428
Number of obs: 2000, groups:  sub, 100; item, 20

Fixed effects:
                      Estimate Std. Error         df t value Pr(>|t|)
(Intercept)            1.65600    0.06598  255.33830  25.100  < 2e-16 ***
con1c2                 0.30800    0.06644 1899.99429   4.636 3.79e-06 ***
con2c2                -0.05600    0.06644 1899.99429  -0.843   0.3994
dysd2                 -0.20800    0.09330  255.33830  -2.229   0.0267 *
con1c2:con2c2         -0.08800    0.09396 1899.99429  -0.937   0.3491
con1c2:dysd2          -0.09600    0.09396 1899.99429  -1.022   0.3070
con2c2:dysd2          -0.10400    0.09396 1899.99429  -1.107   0.2685
con1c2:con2c2:dysd2    0.20800    0.13287 1899.99429   1.565   0.1177
---
Signif. codes:  0 '***' 0.001 '**' 0.01 '*' 0.05 '.' 0.1 ' ' 1

Correlation of Fixed Effects:
            (Intr) con1c2 con2c2 dysd2  cn12:22 cn12:2 cn22:2
con1c2      -0.503
con2c2      -0.503  0.500
dysd2       -0.707  0.356  0.356
cn1c2:cn2c2  0.356 -0.707 -0.707 -0.252
cn1c2:dysd2  0.356 -0.707 -0.354 -0.503  0.500
cn2c2:dysd2  0.356 -0.354 -0.707 -0.503  0.500   0.500
cn1c2:c22:2 -0.252  0.500  0.500  0.356 -0.707  -0.707 -0.707
convergence code: 0
boundary (singular) fit: see ?isSingular
```

Working Memory

```
Linear mixed model fit by maximum likelihood . t-tests use Satterthwaite's method ['lmerModLmerTest']
Formula: reg ~ con1 * con2 * dys * wm + (1 | sub) + (1 | item)
   Data: paul

     AIC      BIC   logLik deviance df.resid
  4673.8   4780.3  -2317.9   4635.8     1981

Scaled residuals:
    Min      1Q  Median      3Q     Max
-2.7667 -0.7387  0.0272  0.6269  4.0444

Random effects:
 Groups   Name        Variance  Std.Dev.
 sub      (Intercept) 1.032e-01 3.212e-01
 item     (Intercept) 1.128e-16 1.062e-08
 Residual             5.500e-01 7.416e-01
Number of obs: 2000, groups:  sub, 100; item, 20

Fixed effects:
                         Estimate Std. Error         df t value Pr(>|t|)
(Intercept)             1.753e+00  1.536e-01  2.607e+02  11.410  < 2e-16 ***
con1c2                  4.058e-01  1.560e-01  1.900e+03   2.601  0.00937 **
con2c2                  9.156e-02  1.560e-01  1.900e+03   0.587  0.55741
dysd2                  -1.129e-01  2.323e-01  2.607e+02  -0.486  0.62736
wm                     -5.698e-03  8.207e-03  2.607e+02  -0.694  0.48813
con1c2:con2c2          -4.452e-01  2.207e-01  1.900e+03  -2.018  0.04378 *
con1c2:dysd2           -2.682e-01  2.360e-01  1.900e+03  -1.137  0.25581
con2c2:dysd2           -3.674e-01  2.360e-01  1.900e+03  -1.557  0.11967
con1c2:wm              -5.775e-03  8.337e-03  1.900e+03  -0.693  0.48862
con2c2:wm              -8.711e-03  8.337e-03  1.900e+03  -1.045  0.29625
dysd2:wm               -5.116e-03  1.227e-02  2.607e+02  -0.417  0.67696
con1c2:con2c2:dysd2     8.618e-01  3.337e-01  1.900e+03   2.582  0.00989 **
con1c2:con2c2:wm        2.109e-02  1.179e-02  1.900e+03   1.788  0.07387 .
con1c2:dysd2:wm         9.974e-03  1.246e-02  1.900e+03   0.800  0.42360
con2c2:dysd2:wm         1.525e-02  1.246e-02  1.900e+03   1.223  0.22130
con1c2:con2c2:dysd2:wm -3.783e-02  1.762e-02  1.900e+03  -2.146  0.03198 *
---
Signif. codes:  0 '***' 0.001 '**' 0.01 '*' 0.05 '.' 0.1 ' ' 1

Correlation matrix not shown by default, as p = 16 > 12.
Use print(x, correlation=TRUE)  or
    vcov(x)        if you need it

convergence code: 0
boundary (singular) fit: see ?isSingular
```

Working Memory – Dyslexics

```
Linear mixed model fit by maximum likelihood . t-tests use Satterthwaite's method ['lmerModLmerTest']
Formula: reg ~ con1 * con2 * wm + (1 + con1 | sub) + (1 | item)
   Data: paul

     AIC      BIC   logLik deviance df.resid 
  2343.1   2406.9  -1158.5   2317.1      987 

Scaled residuals: 
    Min      1Q  Median      3Q     Max 
-2.8326 -0.7778  0.0123  0.5614  4.0817 

Random effects:
 Groups   Name        Variance Std.Dev. Corr
 sub      (Intercept) 0.03940  0.1985       
          con1c2      0.03312  0.1820   1.00
 item     (Intercept) 0.00000  0.0000       
 Residual             0.55202  0.7430       
Number of obs: 1000, groups:  sub, 50; item, 20

Fixed effects:
                   Estimate Std. Error         df t value Pr(>|t|)    
(Intercept)        1.752518   0.128759 151.169975  13.611   <2e-16 ***
con1c2             0.405824   0.167640 257.186189   2.421   0.0162 *  
con2c2             0.091560   0.156324 950.000618   0.586   0.5582    
wm                -0.005698   0.006880 151.169975  -0.828   0.4089    
con1c2:con2c2     -0.445211   0.221076 950.000619  -2.014   0.0443 *  
con1c2:wm         -0.005775   0.008957 257.186189  -0.645   0.5197    
con2c2:wm         -0.008711   0.008353 950.000617  -1.043   0.2973    
con1c2:con2c2:wm   0.021087   0.011813 950.000619   1.785   0.0746 .  
---
Signif. codes:  0 '***' 0.001 '**' 0.01 '*' 0.05 '.' 0.1 ' ' 1

Correlation of Fixed Effects:
            (Intr) con1c2 con2c2 wm     cn12:22 cn1c2: cn2c2:
con1c2      -0.381                                           
con2c2      -0.607  0.466                                    
wm          -0.905  0.345  0.549                             
cn1c2:cn2c2  0.429 -0.659 -0.707 -0.389                      
con1c2:wm    0.345 -0.905 -0.422 -0.381  0.597               
con2c2:wm    0.549 -0.422 -0.905 -0.607  0.640   0.466       
cn1c2:cn22: -0.389  0.597  0.640  0.429 -0.905  -0.659 -0.707
convergence code: 0
boundary (singular) fit: see ?isSingular
```

Working Memory – Controls

```
Linear mixed model fit by maximum likelihood . t-tests use Satterthwaite's method ['lmerModLmerTest']
Formula: reg ~ con1 * con2 * wm + (1 + con1 | sub) + (1 | item)
   Data: paul

     AIC      BIC   logLik deviance df.resid
  2326.3   2390.1  -1150.1   2300.3      987

Scaled residuals:
    Min      1Q  Median      3Q     Max
-2.6430 -0.6992 -0.0055  0.6949  3.2188

Random effects:
 Groups   Name        Variance Std.Dev. Corr
 sub      (Intercept) 0.088805 0.29800
          con1c2      0.011643 0.10790  1.00
 item     (Intercept) 0.002909 0.05393
 Residual             0.532705 0.72987
Number of obs: 1000, groups:  sub, 50; item, 20

Fixed effects:
                   Estimate Std. Error         df t value Pr(>|t|)
(Intercept)        1.641794   0.167748 101.285652   9.787 2.57e-16 ***
con1c2             0.132400   0.180651 431.738950   0.733   0.4640
con2c2            -0.280164   0.174450 944.236129  -1.606   0.1086
wm                -0.010936   0.008732 100.335434  -1.252   0.2133
con1c2:con2c2      0.426982   0.246710 944.236127   1.731   0.0838 .
con1c2:wm          0.004492   0.009368 472.337743   0.479   0.6318
con2c2:wm          0.006781   0.009130 945.719125   0.743   0.4578
con1c2:con2c2:wm  -0.017324   0.012911 945.719123  -1.342   0.1800
---
Signif. codes:  0 '***' 0.001 '**' 0.01 '*' 0.05 '.' 0.1 ' ' 1

Correlation of Fixed Effects:
            (Intr) con1c2 con2c2 wm     cn12:22 cn1c2: cn2c2:
con1c2      -0.360
con2c2      -0.520  0.483
wm          -0.922  0.327  0.485
cn1c2:cn2c2  0.368 -0.683 -0.707 -0.343
con1c2:wm    0.328 -0.919 -0.452 -0.355  0.639
con2c2:wm    0.482 -0.448 -0.927 -0.523  0.656   0.487
cn1c2:cn22: -0.341  0.633  0.656  0.370 -0.927  -0.689 -0.707
convergence code: 0
boundary (singular) fit: see ?isSingular
```

Verbal Intelligence

```
Linear mixed model fit by maximum likelihood . t-tests use Satterthwaite's method ['lmerModLmerTest']
Formula: reg ~ con1 * con2 * dys * viq + (1 | sub) + (1 | item)
   Data: paul

     AIC      BIC   logLik deviance df.resid
  4675.7   4782.2  -2318.9   4637.7     1981

Scaled residuals:
    Min      1Q  Median      3Q     Max
-2.7959 -0.7497  0.0418  0.5993  4.1450

Random effects:
 Groups   Name        Variance Std.Dev.
 sub      (Intercept) 0.1064   0.3262
 item     (Intercept) 0.0000   0.0000
 Residual             0.5498   0.7415
Number of obs: 2000, groups:  sub, 100; item, 20

Fixed effects:
                          Estimate Std. Error         df t value Pr(>|t|)
(Intercept)              1.650e+00  6.655e-02  2.560e+02  24.801  < 2e-16 ***
con1c2                   2.968e-01  6.709e-02  1.900e+03   4.424 1.02e-05 ***
con2c2                  -5.314e-02  6.709e-02  1.900e+03  -0.792   0.4285
dysd2                   -1.982e-01  9.413e-02  2.560e+02  -2.106   0.0362 *
viq                      3.668e-02  6.618e-02  2.560e+02   0.554   0.5799
con1c2:con2c2           -6.614e-02  9.488e-02  1.900e+03  -0.697   0.4858
con1c2:dysd2            -7.945e-02  9.491e-02  1.900e+03  -0.837   0.4026
con2c2:dysd2            -1.091e-01  9.491e-02  1.900e+03  -1.149   0.2507
con1c2:viq               7.340e-02  6.672e-02  1.900e+03   1.100   0.2715
con2c2:viq              -1.885e-02  6.672e-02  1.900e+03  -0.283   0.7775
dysd2:viq               -9.134e-03  9.463e-02  2.560e+02  -0.097   0.9232
con1c2:con2c2:dysd2      1.849e-01  1.342e-01  1.900e+03   1.378   0.1684
con1c2:con2c2:viq       -1.439e-01  9.436e-02  1.900e+03  -1.525   0.1275
con1c2:dysd2:viq        -3.785e-02  9.541e-02  1.900e+03  -0.397   0.6916
con2c2:dysd2:viq         4.462e-03  9.541e-02  1.900e+03   0.047   0.9627
con1c2:con2c2:dysd2:viq  1.359e-01  1.349e-01  1.900e+03   1.008   0.3138
---
Signif. codes:  0 '***' 0.001 '**' 0.01 '*' 0.05 '.' 0.1 ' ' 1

Correlation matrix not shown by default, as p = 16 > 12.
Use print(x, correlation=TRUE)  or
    vcov(x)        if you need it

convergence code: 0
boundary (singular) fit: see ?isSingular
```

Section G: Analysis of reading times, regressions, and comprehension comparing correct and incorrect trials.

In the tables below, we present the correlations between eye movement measures and comprehension accuracy.

Table S1 presents correlations between total sentence reading time and comprehension, and Table S2 presents correlations between regressions and comprehension. Results are provided for the full sample and also broken down by the two groups (control and dyslexics). In addition, in Table S3 we have also provided the means for correct and incorrect responses separately for controls and dyslexics.

Table S1. *Bivariate correlations between comprehension accuracy and reading time.*

	Full Sample	Controls	Dyslexics
Active-plausible	-.08	-.04	-.06
Active-implausible	-.02	.03	-.04
Passive-plausible	.02	.01	.04
Passive-implausible	-.01	.05	.02

Note. $^{\#}p < .08$, $^{*}p < .05$, $^{**}p < .01$.

Table S2. *Bivariate correlations between comprehension accuracy and regressions.*

	Full Sample	Controls	Dyslexics
Active-plausible	-.03	-.02	.11
Active-implausible	.06	.02	.01
Passive-plausible	.05	.07	.03
Passive-implausible	.03	.08	.02

Note. $^{\#}p < .08$, $^{*}p < .05$, $^{**}p < .01$.

Table S3. *Mean reading times (msec) and regressions for correct and incorrect responses by group and experimental condition.*

	Incorrect Reading times	Incorrect Regressions	Correct Reading times	Correct Regressions
Controls				
Active-implausible	2739	1.41	2838	1.45
Active-plausible	2608	1.36	2420	1.28
Passive-implausible	1908	1.52	2008	1.69
Passive-plausible	2016	1.43	2038	1.65
Dyslexics				
Active-implausible	3545	1.49	3381	1.69
Active-plausible	3327	1.58	3069	1.60
Passive-implausible	2658	1.93	2724	1.98
Passive-plausible	2361	1.75	2538	1.83
